# Influence of Blanching Time on the Phytochemical and Nutritive Value of Cowpea (*Vigna unguiculata* L. Walp) Leafy Vegetable

**DOI:** 10.1155/2024/9095035

**Published:** 2024-07-29

**Authors:** M. Y. Maila, P. E. Tseke

**Affiliations:** ^1^ Limpopo Agro-Food Technology Station University of Limpopo, Private Bag x1106, Sovenga 0727, South Africa; ^2^ Green Biotechnologies Research Centre of Excellence University of Limpopo, Private Bag x1106, Sovenga 0727, South Africa

## Abstract

Seasonal production limits the effective utilization of cowpea, which is regarded as food and a cash income crop in most African rural communities. To reduce the bacterial content and inactivate the naturally occurring enzymes that could induce undesirable changes during storage of the vegetable, blanching is applied. However, loss in flavor and nutritional value is experienced. Therefore, a study was conducted to investigate the effects of different blanching times on cowpea at a constant temperature, on its nutritive value. A 2 kg sample of fresh leaves was divided into four portions and blanched at 95°C for 0 (control), 2.5, 5, and 10 min. The study was arranged in a completely randomized design in triplicate. Collected data included moisture, total phenolic, antioxidant activity, and minerals [calcium (Ca), potassium (K), cobalt (Co), chromium (Cr), and silicon (Si)]. Compared to the control, total phenolic increased at 2.5 min to 9.83 mg GAE/g but then decreased by 4.99 and 4.60 mg GAE/g at 5 and 10 min of blanching time, respectively. Similarly, antioxidant activity increased at 2.5 min to 1025 *μ*g AAE/g of WM, but reduced by 751.71 and 641.80 *μ*g AAE/g of WM at 5 and 10 min, respectively. Ca increased at 2.5, 5, and 10 min by 69.10, 62.47, and 74.53 mg/L, respectively. Similarly, K increased at 2.5, 5, and 10 min by 31.57, 49.13, and 46.03 mg/L, respectively. Contrarily, Co decreased by 7.65, 7.37, and 9.29 mg/L at 2.5, 5, and 10 min of blanching, respectively. Similarly, Cr also decreased at 2.5, 5, and 10 min, by 0.23, 0.35, and 0.56 mg/L, respectively. Si increased at 2.5 and 10 min by 4.15 and 3.31 mg/L and reduced at 5 min by 1.61 mg/L. Therefore, blanching time of 2.5 min at a constant temperature of 95°C increased the tested nutritive elements, except for moisture, Co, and Cr.

## 1. Introduction

The cowpea (*Vigna unguiculata* (L.) Walp) leafy vegetable in the Fabaceae family is indigenous to Africa [1]. Generally, both grains and tender leaves serve as a major source of food security in many African households especially in rural communities. Subsistence and backyard farmers are the major producers of cowpea leafy vegetable and its grains under dryland farming conditions, especially during summer rainfall seasons. Generally, cowpea grain production is estimated at 6.5 million tons per annum on 14.5 million hectares, worldwide [2]. In some parts of the African continent, the cowpea grains are widely consumed as rich sources of protein and other nutrients, and in South Africa (SA), the tender leaves of cowpea are consumed as a green leafy vegetable and have been proven to be a good source of vitamins and minerals [1].

Nutritionally, the tender leaves are a rich source of proteins; minerals, such as calcium (Ca), phosphorus (P), and iron (Fe); and vitamins such as provitamin A, folate, thiamin, riboflavin, and vitamin C [3, 4]. In a trial on displaced people, findings indicated that 6 tonnes of tender cowpea leaves, which is equivalent to a tonne of dried cowpea leaves, could supply about 800 children aged 4–6 years old with 10 g leaf concentrate that can provide them with 7.75 mg of iron (Fe) per week all year round.

Even with the known nutraceutical value of the crop, Kirakou et al. [4] documented that the most limiting factor to the efficient production and utilization of cowpea leaves is the seasonality in their production. Its production and utilization is mainly seasonal as a result of a myriad of challenges, which includes limited postharvest handling technologies [5]. During the glut season, high postharvest losses are experienced due to the limited postharvest technologies, whereas during drought, there is scarcity [6]. Generally, many subsistence cowpea producers still lack appropriate storage and postharvest methods that would ensure its availability during off-season. As a result, some local communities developed the traditional value-addition processing techniques (blanching and sun-drying) aimed at enhancing the storage and availability of the leafy vegetables even during seasons of drought [7].

In a study by Mokganya, Mushaphi, and Tshisikhawe [8] on wild vegetables use by the indigenous Vhavenda tribe in the Venda region of Limpopo Province, boiling and sun-drying were their preferred methods of preserving the vegetables. The reason for preserving the vegetables was that they serve as food during times of droughts. However, in traditional boiling method, the initial water is often discarded few minutes after boiling the vegetables to reduce undesirable bitterness, colors, and flavor, which in turn affects the nutritional quality of the desired leafy vegetable. Generally, being aware of the necessary procedures required in the preparation, preservation, and storage of leafy vegetables, could bring consumer awareness on how to get maximum nutrition and flavors [9].

Generally, blanching is the process of soaking vegetables in hot water for a short period of time in order to preserve the flavor, color, texture, and nutritional value. However, Njoroge et al. [10] reported that variation in the blanching temperature–time combination has an effect on the nutritional composition and microbial quality of the blanched leafy vegetable. Major losses of important nutrients, namely, *β*-carotene and vitamin C up to 55.5% and 61.1%, respectively, were documented for cowpea leafy vegetables that were subjected to blanching in a pressure cooker at 121°C for 15 min [11]. Therefore, this study objective was to investigate the effects of blanching time–temperature on the nutritive value of cowpea leafy vegetable.

## 2. Materials and Methods

### 2.1. Sample Collection and Study Site

Tender leaves of cowpea were harvested from the cultivated cropland at the Green Biotechnology Research Centre of Excellence (GBRCE) at the University of Limpopo (23°53′10^″^ S, 29°44′15^″^ E), Limpopo Province of SA. The location at GBRCE has hot and dry summers (Nov–Jan), with daily maximum temperatures ranging from 28°C to 38°C. The average annual rainfall had previously been less than 500 mm, with the distribution skewed towards summers. The area had a slope of 0.05%, containing Hutton soil with loamy soil (65% sand, 30% clay, and 5% silt), 1.6% organic C, and ECe 0.148 dS/m [12, 13]. The freshly harvested leaves were kept in brown paper bags and stored in a cooler box to preserve their freshness in transit to Limpopo Agro-Food Technology Station (LATS) for further processing and analysis. The study was then conducted at LATS, at the University of Limpopo (23°53′10^″^ S, 29°44′15^″^ E), Limpopo Province of SA.

### 2.2. Cowpea Sample Preparation and Blanching Procedure

The harvested cowpea leafy vegetable samples were first sorted to remove damaged leaves, washed with tap water to remove dust on the leaf surface, drained, shredded, and then weighed to obtain sample mass before blanching. A 2-kg sample was divided into four equal portions and blanched at a constant temperature of 95°C for 0, 2.5, 5, and 10 min. This was achieved by bringing a large stainless steel of thermostatically regulated hot water bath (Gallenkamp, United Kingdom) half-full of water to 95°C. Then, the shredded vegetables were put into a wire mesh basket and gently lowered into the blanching hot water. At the end of each blanching period, the baskets with vegetables were removed from the boiling water and plunged into cold water at room temperature to stop the blanching process. The blanched leaves were drained off the water and cooled in a laboratory room temperature (20°C–22°C) temperature for 5 min [10]. The blanched samples were air-dried at room temperature until excess water was removed, then ground to a fine powder using a microfine grinder (IKA ® MF 10 basic, Werke), and stored in zip-locked plastic bags in preparation for nutritional analysis.

### 2.3. Study Design and Treatments

The study was arranged in a complete randomized design (CRD) in triplicate. The treatments comprised four different blanching times, namely, 0 (control), 2.5, 5, and 10 min, at a constant temperature of 95°C.

### 2.4. Nutrient Determination and Data Collection

Moisture content was determined using a moisture analyzer (Max 50, RADWAG). A constant mass of 0.2 g of each sample was loaded into a weighing chamber and heated at 121°C on the moisture analyzer until a constant mass was achieved. The mass at drying was recorded as the moisture content of the sample [14]. Results were expressed as percentages. Total phenolic content was determined using Adetutu, Olorunnisola, and Owoade [15] method with minimal modification. A reaction was established by treating 0.250 mL of methanolic extract with 2.5 mL of Folin–Ciocalteu reagent (2 N) for 5 min. The reaction was stopped by adding 7.5 mL of 20% anhydrous sodium carbonate. The volume was made up to 50 mL with distilled water, and it was then incubated for 2 h at room temperature in the dark. After incubation, the absorbance was read at 760 nm with a spectrometer (GENESYS 20, Thermo Spectronic). The phenolic content was determined from a standard curve of different concentrations of garlic acid. The results were expressed as milligram of garlic acid equivalent/gram (mg GAE/g) of wet material.

The antioxidant content of the blanched samples was determined using a phosphomolybdate antioxidant assay according to Prabhavathi, Prasad, and Jayaramu [16]. Phosphomolybdate reagent was prepared by mixing equal amounts of stock solutions of 0.6 M sulphuric acid, 4 mm ammonium molybdate, and 28 mm sodium phosphate. The reaction was initiated by mixing 3 mL of phosphomolybdate reagent with 300 *μ*L of methanolic extract or standard solution or methanol in a test tube and mixing. The test tube was covered with foil, incubated at 95°C for 90 min, and then allowed to cool to room temperature. The absorbance of the green-colored contents was measured at 695 nm against a blank. Ascorbic acid (AA) was used as a standard, and results were expressed as micrograms per millilitre of AA equivalents (*μ*g AAE/g of WM).

For mineral determination, the wet digestion method was used according to Wheal and Palmer [17] with modifications. The analyzed minerals were Ca, potassium (K), cobalt (Co), chromium (Cr), and silicon (Si), which were expressed as milligrams per liter. About 0.2 g of the blanched samples was digested with 40 mL of 5% nitric acid for 60 min at 95°C. The solutions were filtered using Whatman No. 1 filter paper and then covered with aluminum foil. Mineral analysis was performed using a multitype Shimadzu inductively coupled plasma emission spectrophotometer (ICPE-9000). Standard solutions of each mineral were used for the calibration of the instrument.

### 2.5. Data Analysis

All data were tested for normality of distribution using the Shapiro–Wilk test [18, 19] prior to analysis of variance (ANOVA) using SAS software [20]. When the treatments were significant at the probability level of 5%, the degree of freedom and their associated mean sum of squares were partitioned to determine the percentage contribution of sources of variation to total treatment variation (TTV) among the treatment means. At a probability level of 5%, mean separation was achieved using Fisher's least significant difference (LSD) test. Bar charts were also used to show the differences. Graphs utilize standard error bars to show the uncertainty or error in a given measurement. The results were given and discussed at a 5% level of significance.

## 3. Results

**Figure 1:**
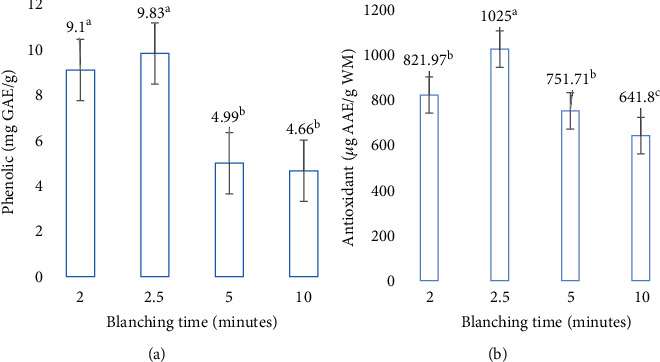
Response of (a) total phenolic (mg GAE/g) and (b) antioxidant contents (*μ*g AAE/g WM) of cowpea leafy vegetable blanched at different times. Graphs utilize standard error bars to show the uncertainty. ± standard error followed by the same letter was not significantly different at *p* ≤ 0.05 according to Fisher's least significant difference (LSD) test.

Blanching time had no significant (*p* ≤ 0.05) effects on the moisture content of cowpea leafy vegetable, although higher significant (*p* ≤ 0.01) effects were observed in the total phenolic and antioxidant content of the vegetable (Table 1). In terms of moisture, phenolic, and antioxidant content, treatments contributed 44%, 95%, and 97% in TTV, respectively (Table 1). Total phenolic increased at 2.5 min of blanching time to 9.83 mg GAE/g and then decreased at 5 and 10 min of blanching to 4.99 and 4.66 mg GAE/g, respectively, when compared to the control (Figure 1(a)). Similarly, relative to the control, antioxidant activity increased at 2.5 min blanching time to 1025 *μ*g AAE/g WM and then reduced at 5 and 10 min of blanching time to 751.71 and 641.8 *μ*g AAE/g WM, respectively (Figure 1(b)).

**Figure 2:**
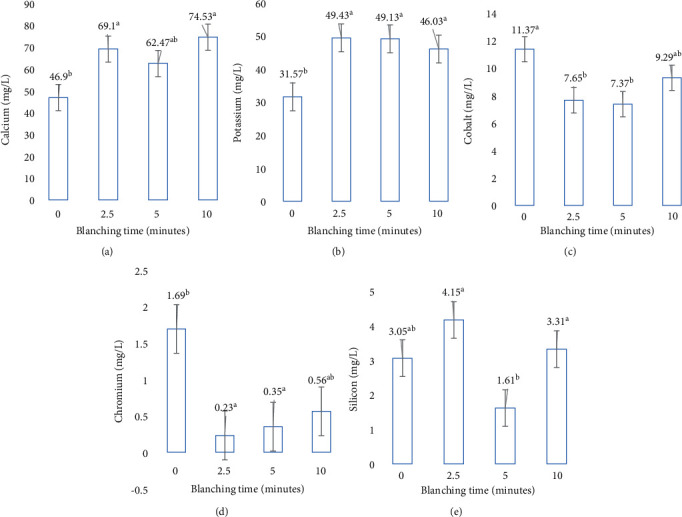
Response of (a) calcium, (b) potassium, (c) cobalt, (d) chromium, and (e) silicon (milligrams per liter) contents of cowpea leafy vegetable blanched at different times. Graphs utilize standard error bars to show the uncertainty. ± standard error followed by the same letter was not significantly different at *p* ≤ 0.05 according to Fisher's least significant difference (LSD) test.

In terms of the mineral content analysis, results showed that blanching time had significant (*p* ≤ 0.05) effects on Ca, K, Co, Cr, and Si content of the leafy vegetable, contributing 85%, 94%, 82%, 83%, and 82% of TTV, respectively (Table 2). Relative to the control, Ca element increased at 2.5, 5, and 10 min of blanching time to 69.1, 62.47, and 74.53 mg/L, respectively (Figure 2(a)). Similarly, K in blanched cowpea leaves increased at 2.5, 5, and 10 min blanching time to 31.57, 49.13, and 46.03 mg/L, respectively (Figure 2(b)). In contrast, relative to the unblanched control, Co decreased at 2.5, 5, and 10 min, to 7.65, 7.37, and 9.29 mg/L, respectively (Figure 2(c)). Similarly, Cr decreased at 2.5, 5, and 10 min, to 0.23, 0.35, and 0.56 mg/L, respectively (Figure 2(d)). On the other hand, when compared to the unblanched leaves at the control, Si increased at 2.5 and 10 min of blanching time to 4.15 and 3.31 mg/L but significantly reduced at 5 min to 1.61 mg/L (Figure 2(e)).

## 4. Discussion

Among the other preservative methods of indigenous leafy vegetables, blanching has been used to preserve cowpea leafy vegetables and extend their utilization shelf life. Even in their preserved forms, cowpea leaves are known to be rich in nutrients that are essential for the normal functioning of the human body. The nonsignificant effects of blanching time on the leaf moisture content observed in this study were in line with the findings from Natabirwa et al. [21], who observed nonsignificant differences in the moisture content and other proximate macronutrient compositions of cleome (Cleome gynandra) leafy vegetables under different blanching time. Higher significant effects in the total phenolic and antioxidant content of the vegetable attained were in agreement with findings by Adjei-Fremah, Jackai, and Worku [22] who confirmed similar observations, indicating that the highly significant effects on cowpea leaves symbolize the high amounts of total phenolic and antioxidant content in the leaves.

The observed increase in total phenolic content at 2.5 min of blanching time compared with 0, 5, and 10 min of blanching time shows that blanching cowpea leaves is necessary for increasing total phenolic content in leaves. However, a shorter blanching duration (2.5 min) is most appropriate, as longer durations lead to substantial decreases in total phenolic contents in leaves. The increase in total phenolic content at 2.5 min of blanching time correlates the findings by Sheetal, Jyothi, and Jamuna [23] and the report of Liu et al. [24], where it was opined that cooling or blanching could increase phenol contents in vegetables. According to Bamidele et al. [25], the increase in the total phenolic content could be attributed to the reduction of enzyme-mediated polyphenol degradation (complete inactivation of native polyphenol oxidase). Furthermore, the observed increase could also be due to the release of bound phenolic acids from the breakdown of cellular constituents of the plant cell walls in the leafy vegetable [25].

Contrarily, the loss of total phenolic at 5 and 10 min of blanching time may be attributed to diffusion and leaching. Negi and Roy [26] documented that the reduction might also be due to the degradation of phenolic compounds by heat or their leaching out from the vegetable tissues into the blanching water. Similarly, Irondi et al. [27] observed a significant losses of both the flavonoids (except quercetin) and phenolics in the leaves of *Adansonia digitata* at 10 min blanching time. Sheetal, Jyothi, and Jamuna [23] recommended that the length of the blanching time in green leafy vegetables should be between 1 and 5 min. Various recommendations on the blanching time suggest a short holding time, which may stimulate enzyme actions instead of denaturing [10]. Exposing entities to shorter processing times encourage smaller losses of vitamins and other soluble heat-sensitive constituents of food, which support the observation from the current study. Francisco et al. [28] stated that some phenolic compounds are known to be in soluble form in combination with the plant cell wall components. However, Bamidele et al. [25] reported that during blanching (high temperature of 90°C and time 10–15 min), the disruption of the plant cell wall may occur leading to leaching out of the soluble phenolic compound.

The antioxidant activities of the cowpea-blanched leaf samples ranged between 641.8 and 1025 *μ*g AAE/g at 0–10 min in this study. At 2.5 min, the scavenging power of the vegetable extracts increased with blanching time. Incidentally, the same scavenging power of the vegetable extracts was reduced when the blanching time was increased at 5–10 min. Several authors have observed similar trends of decrease in the radical scavenging power of blanched leafy vegetables [29–31]. The documented reduction is mostly attributed to the decrease in the radical scavenging power to the loss of water-soluble and thermal degradation of phenolic compounds during blanching. Reblova [32] opined that the decrease in antioxidant activity with high temperature is generally described by the acceleration of commencement responses connected with faster exploitation of antioxidants. The results observed are in line with Njoroge et al. [10], who reported that variation in blanching time and temperature results in various effects on the nutritional value of the vegetables.

The blanched cowpea leafy vegetables accumulated higher Ca and K mineral contents compared to the untreated control. The increase in the Ca contents of the blanched cowpea leafy vegetables means that blanching is favorable in maintaining Ca in cowpea leaves. The results are in line with observations by Fadupin et al. [33], in selected green leafy vegetables consumed in Nigeria. Park, Jang, and Lee [34] suggested that oxalate, which occurs naturally in most plants, is available as soluble and insoluble salts with Ca. Almost 30% of the total Ca in vegetables is known to be bound with soluble oxalate, while the remaining 70% exists as insoluble oxalate. However, blanching has been reported to decrease the soluble oxalate content of the vegetables by leaching into water thereby releasing more Ca mineral content within the vegetable [35, 36], which could explain the increase of blanched leaf Ca contents. Brogren and Savage [37] were of the opinion that soluble oxalates were reduced by 66% in the blanched vegetables by leaching into water, thus releasing the bound nutrients.

In contrast, the findings in this study with regard to K mineral content contradicted with results in a study by Fadupin et al. [33]. In their study on selected green leafy vegetables, the K content was high in unblanched form but started decreasing when the vegetable was blanched for 10 min, which in our case, K was still increasing at 10 min blanching time in cowpea leafy vegetable. The increase of K in the current study could be attributed to the fact that the temperature used during blanching was still relatively correlated to enzyme activity found in the blanched vegetable and K did not experience denaturation.

With Cr and Co, the results also showed that the loss in the two minerals increased as the blanching time increased, although the effect varied among the samples. The reduction in the mineral content of all the tested samples after blanching at various times could be attributed to the leaching out of most water-soluble minerals during blanching. Since most of these minerals are present in the cell wall of the plant, disruption of the plant cell wall will leach them out into the water during blanching. It can also be said that the longer the leafy vegetable remains in the water during blanching, the more the reduction in the determined minerals [25, 38].

The finding in this study also indicated that Si increased at 2.5 and 10 min of blanching time, while the mineral content reduced at 5 min of blanching time. Accumulation of Si at 2.5 blanching time could be attributed to the activation of enzymes or acids, which is responsible for the trickery of the silicic acid (Si(OH)_4_), in either both passive and active forms. During the early stages of exposure to low temperatures with low time, generally, cellular respiration increases. Although temperatures above 60°C denature enzymes, which are responsible to drive the secondary metabolite progressions in thermo-unstable chemical compounds. High temperatures increase the volatilization of substances, thus reducing the concentrations of directed vigorous elements. In a study by Pereira et al. [39], results showed that cowpea leaves accumulated a high levels of Si due to the feasible strategy used to avoid cadmium (Cd) toxicity, which is currently unidentified. The reduction of Si at 5 min could be attributed to chemical degradation with associated high temperatures, which depends on the chemical bonds within the chemical compounds.

## 5. Conclusions

In conclusion, the use of blanching as a preservative method for cowpea leafy vegetable had great effects on total phenolic and antioxidant activities. At 2.5 min blanching time at a constant temperature of 95°C, total phenolic and antioxidant activity were enhanced. The mineral contents, namely, Ca, K, and Si, were also influenced by blanching time, except for Co and Cr, which were reduced with blanching time. Reduced mineral contents were achieved when more blanching time was used. Therefore, it is recommended to consumers and the food industry to blanch cowpea leafy vegetable at 2.5 min at a constant temperature of 95°C for enhanced nutritive value. Further blanching studies are encouraged in order to perform the complete nutritional analysis excluded in the study.

## Figures and Tables

**Table 1:** Partitioning of mean sum of squares (MSS) for moisture (%), total phenolic (mg GAE/g), and antioxidant (*μ*g AAE/g) contents of cowpea leafy vegetable blanched at different times.

**Source**	**DF**	**Moisture (%)**		**Total phenolic (mg GAE/g)**		**Antioxidant (*μ*g AAE/g WM)**	
		**MSS**	**%**	**MSS**	**%**	**MSS**	**%**
Treatment	3	53.62	44^ns^	22.21	95^∗∗∗^	78049.0	97^∗∗∗^
Error	8	68.82	56	1.26	5	2640.3	3
Total	11	122.44	100	23.47	100	80689.3	100

Abbreviation: DF = degrees of freedom.

^∗∗∗^Significant at *p* ≤ 0.01.

^ns^Not significant at *p* ≤ 0.05.

**Table 2:** Partitioning of mean sum of squares (MSS) for calcium (Ca), potassium (K), cobalt (Co), chromium (Cr), and silicon (Si) (milligrams per liter) contents of cowpea leafy vegetable blanched at different times.

**Source**	**DF**	**Calcium (mg/L)**		**Potassium (mg/L)**		**Cobalt (mg/L)**		**Chromium (mg/L)**		**Silicon (mg/L)**	
		**MSS**	**%**	**MSS**	**%**	**MSS**	**%**	**MSS**	**%**	**MSS**	**%**
Treatment	3	429.47	85^∗∗∗^	214.59	94^∗∗∗^	0.027	82^∗∗∗^	1.35	83^∗∗∗^	3.352	82^∗∗∗^
Error	8	78.52	15	5.32	6	0.01	18	0.28	17	0.74	18
Total	11	507.99	100	219.91	100	0.03	100	1.62	100	4.09	100

Abbreviation: DF = degrees of freedom.

^∗∗∗^Significant at *p* ≤ 0.05.

## Data Availability

Upon request from the corresponding author, data used to support the findings of this study will be made available for a reason.
